# Low level air pollution and exacerbation of existing copd: a case crossover analysis

**DOI:** 10.1186/s12940-016-0179-z

**Published:** 2016-10-18

**Authors:** Rebecca DeVries, David Kriebel, Susan Sama

**Affiliations:** Department of Work Environment, University of Massachusetts Lowell, 1 University Avenue, Lowell, MA 01854 USA

**Keywords:** Air pollution, Sulfur dioxide, Nitrogen dioxide, Particulate matter, Chronic obstructive pulmonary disease, Case-crossover

## Abstract

**Background:**

Exacerbations of chronic obstructive pulmonary disease (COPD) contribute greatly to increased morbidity, mortality and diminished quality of life. Recent studies report moderately strong positive associations between exposures to several air pollutants and COPD-related emergency department (ED) visits and hospital admissions (HA). Studies that use clinically defined exacerbations rather than counting ED visits and HA may be more sensitive to environmental triggers like air pollution, but very few such studies exist. Participants in a COPD disease management group living in an area of low air pollution and who were followed closely for the earliest signs of an exacerbation provided an opportunity to study associations between air pollution and COPD exacerbation.

**Methods:**

Associations between short term exposures to air pollutants, including sulfur dioxide (SO_2_), nitrogen dioxide (NO_2_), and particulate matter < 2.5 microns (PM_2.5_), and COPD exacerbation were assessed among 168 patients residing in central Massachusetts, a region with air pollution levels well below USEPA National Ambient Air Quality Standards (NAAQS). Case-crossover analyses and multivariate conditional logistic regression were used to estimate associations between 7-day average concentrations of each air pollutant, as measured at central site monitors, and COPD exacerbation experienced in the patients’ homes during the period 2012–2013, while controlling for temperature and self-reported influenza.

**Results:**

We found that short-term exposures to SO_2_ were associated with an increase in COPD exacerbation risk (odds ratio (OR) = 2.45, 95 % CI: 1.75–3.45 per 1 ppb increase) after adjustment for PM_2.5_. Short-term exposures to NO_2_ concentrations showed a weaker association, (OR = 1.17, 95 % CI: 1.05–1.30 per 1 ppb increase) after adjustment for PM_2.5_. An unexpectedly modest negative association was seen for short-term exposures to PM_2.5_.

**Conclusions:**

Despite living in an area with air pollution concentrations below current USEPA NAAQS, these COPD patients appeared to suffer increased risk of COPD exacerbation following short-term exposures to increased concentrations of SO_2_ and NO_2_. An unexpected negative association with PM_2.5_ may result from the complex air chemistry of low level PM in this region.

## Background

Chronic obstructive pulmonary disease (COPD), characterized by irreversible airflow obstruction, is one of the leading causes of morbidity, mortality and health-care costs in the United States. Exacerbations of existing COPD, defined as short-term episodes during which the normal inflammatory process is enhanced [1, 2], contribute greatly to increased morbidity and diminished quality of life among COPD patients, with higher exacerbation frequencies associated with poorer health status, increased hospitalizations, and increased costs [1–3]. In the United States, the estimated direct and indirect costs of COPD are $29.5 billion and $20.4 billion, respectively, with exacerbations accounting for the largest proportion of the total economic burden [1]. The economic burden of COPD is likely to be comparable in other developed countries. Well known risk factors for these acute exacerbations include bacterial and viral infections, as well as exposures to tobacco [2] and some workplace dusts and fumes [1]. Recent literature also suggests that certain weather and air quality conditions may trigger an exacerbation of COPD symptoms [4–6]. COPD exacerbations are studied epidemiologically in a variety of ways; the air pollution literature has tended to count emergency department (ED) visits, hospital admissions (HA), or mortality, while studies of risk factors like infection and alternative disease management strategies have used clinical definitions such as increased respiratory symptoms or increased use of steroids and antibiotics.

USEPA has set National Ambient Air Quality Standards (NAAQS) for six criteria air pollutants considered harmful to public health [7]. Four of these are of particular concern to respiratory health and include particulate matter (PM), sulfur dioxide (SO_2_), nitrogen dioxide (NO_2_), and ozone (O_3_). PM is a complex mixture of solid particles and liquid droplets suspended in air and is primarily produced during combustion of fossil fuels by transportation and stationary sources [8]. NAAQS have been set for two particle size fractions; PM_10_ which includes inhalable particles of 10 micrometers or less, and PM_2.5_ which includes fine inhalable particles of 2.5 micrometers or less. SO_2_ is primarily produced from combustion of fossil fuels by electric utilities and industry, while less so by transportation related sources [9]. NO_2_ typically forms from incomplete combustion of nitrogen-containing chemicals, with the largest sources in the US being mobile sources, electric generating units, and industrial/commercial/residential fuels [10]. O_3_ is created in the atmosphere by chemical reactions between volatile organic compounds (VOCs) and oxides of nitrogen (NO_x_) [7]. Individuals with pre-existing respiratory disease (for example, COPD) are potentially susceptible to low concentrations of these four outdoor air pollutants. This study focuses on short-term exposures to SO_2_, NO_2_, and PM_2.5_; O_3_ was excluded since this pollutant is not measured continuously throughout the year in our study area.

Outdoor air pollutants (PM_2.5_, NO_2_, and SO_2_) are well-established risk factors for COPD exacerbation [1, 4], although most studies evaluating these relationships have been conducted in regions with high levels of outdoor air pollution (such as southern California and Hong Kong). Less is known regarding the association between low levels of these outdoor air pollutants and acute exacerbations of COPD that do not result in EDs or HAs. Two epidemiologic studies were identified in published literature that specifically evaluated outdoor air pollution and COPD exacerbations experienced in the patients’ homes [11, 12]. Both studies were completed in London; one reporting increased and the other reporting decreased risk of exacerbation following exposures at comparable concentrations.

This study quantified the association between short term exposures to low levels of outdoor SO_2_, NO_2_, and PM_2.5_ and COPD exacerbation among a group of patients residing in central Massachusetts over a 15-month study period, using a case crossover study design. A major advantage of this study design was that many of the important potential subject-level confounders that remain constant throughout the study period (i.e., age, gender, housing characteristics, co-morbities, and smoking history) were controlled by design (in a case-crossover study, each subject can be seen as “serving as their own control”) [13, 14]. This study also had the benefit of controlling for a number of potential subject-level confounders that vary over time but have rarely been considered in published literature (i.e., respiratory infections, time spent outdoors, health status prior to the event, and physical activities). This robust control for confounding coupled with our sensitive case definition of COPD exacerbation allowed us to detect effects that may not have been captured in some of the larger time series studies that evaluated COPD-related ED/HA, particularly those in regions with higher levels of air pollution.

## Methods

### Study population

The study population was drawn from a COPD disease management group (DMG), maintained by a large medical practice located in Worcester County, Massachusetts. The city of Worcester, located in central Massachusetts, is the third largest city in New England with a population of approximately 180,000 and an estimated population of 6 million within a 50 mile radius [15]. Outdoor air pollution throughout this region is relatively low, with long term annual average concentrations of all primary pollutants consistently falling well below their respective USEPA NAAQS. All recruited participants had received a physician diagnosis of COPD and had no other lung disease (such as interstitial lung disease or lung cancer), with the exception of asthma. All study materials and protocols were approved by the Reliant Medical Group Institutional Review Board.

### Data collected

The data were gathered in a prospective case crossover study designed to evaluate the relationship between indoor and outdoor environmental triggers and exacerbation of existing COPD. Cases were defined as periods of worsening symptoms, which were confirmed by a nurse to require additional oral steroids or antibiotics. During a 15-month study period (January, 2011 to March, 2012), enrolled participants were instructed to call clinic nurses when, following their prescribed DMG treatment plan, they had begun to use their pre-filled medications to treat worsening respiratory symptoms. On this telephone call, a nurse confirmed whether the patient was in fact experiencing an exacerbation and then asked a standard series of questions concerning the patient’s daily living behaviors and activities in the previous week, referred to here as the “exposures questionnaire”. These data, collected at the time of exacerbation (or shortly after), represented the participants’ case periods. The same exposures questionnaire was also administered via telephone at one to three randomly identified times when the participants were not experiencing symptoms of an exacerbation. These data, collected during “healthy weeks”, represented the participants’ control periods. A baseline questionnaire was also administered upon recruitment to gather information on demographics, health status, smoking history, occupational history and housing characteristics.

This paper reports findings of associations between COPD exacerbation and exposures to outdoor air pollutants; a second paper covers results of investigations of household exposures, and activities of daily living. As discussed further below, this division was feasible because we found no evidence of confounding or effect modification of the air pollution risks by any of these risk factors. Temperature and humidity, and colds and flu exposures were also investigated and are discussed here.

### Exposure data: air pollution, weather and influenza

Real-time continuous outdoor air measurements, reported as hourly averages, were available from USEPA’s Air Quality System (AQS) for SO_2_, NO_2_ and PM_2.5_ at the nearby Worcester (EPA monitor # 025-027-0023), Ware (EPA monitor #025-014-4002) and Springfield (EPA monitor # 025-013-0016) central site monitors [16]. 24-h daily average concentrations were extracted for each pollutant and monitor combination. PM_2.5_ data were available at all three monitors as 24-h mass based and 1-h continuous measurements. For this study, we chose to use continuous monitoring data for PM_2.5_ because it resulted in the richest dataset and is consistent with recent air pollution studies. However, since there is some debate regarding the accuracy of these types of continuous measurements, we verified that continuous measurements were appropriate to use for our study period and locations with USEPA’s PM_2.5_ Continuous Monitor Comparability Assessment online application, prior to this decision [17].

Data for important meteorological variables (i.e., temperature and humidity) were exported as 24-h daily averages from the National Oceanic and Atmospheric Administration’s (NOAA) National Climatic Data Center (NCDC) for the Worcester Regional Airport meteorological site [18]. Data on regional trends of influenza were obtained from the US Outpatient Influenza-like Illness Surveillance Network (IliNet), available from the Center for Disease Control’s (CDC) FluView application [19]. The percent of weekly outpatient visits, recorded across all of New England, for influenza-like-illness (defined as fever (temperature of 100 °F or greater), cough, and/or sore throat) was extracted and served as a proxy for regional flu trends. Self-reports of respiratory infections in the prior week were also available from the exposures questionnaire, and we compared the two sources of cold/flu data.

### Statistical analysis

Associations between short-term exposures to outdoor air pollution and COPD exacerbation were quantified with conditional logistic regression in SAS, Version 9.3. Exacerbations were first modeled for single pollutants, with each pollutant serving as the main explanatory variable and COPD exacerbation as a dichotomous outcome (conditional on the individual subject IDs), and then for various combinations of multiple pollutants. Model building was accomplished by adding new variables into the regression model manually based on the −2 log likelihood (−2LL) and strength of association. Main effect estimates were expressed as odds ratios (OR) for a one micro per cubic meter (ug/m^3^) or part per billion (ppb) increase in pollutant concentration.

Pollutant concentrations were modeled as the one-to-seven-day average of daily 24-h average concentrations, as reported in Worcester, Ware, and Springfield, MA. This spatial-temporal exposure metric was selected after completing a sensitivity analysis which investigated single day lags (up to a maximum of seven days) and multi-day averages (0–1 days, 1–2 days, 1–3 days, 1–5 days and 1–7 days) using various combinations of the three monitors [4]. In brief, concentrations were averaged across all three monitors because this aggregation of data provided the best fitting models and the strongest effect estimates. This approach is also consistent with the residential location of study participants and typical east-to-west wind patterns observed in this region [18], as well as supported in the USEPA Integrated Science Assessment document for PM [8]. Daily concentrations were averaged across the seven previous days because this metric resulted in the best fitting models, strongest effect estimates and was consistent with our exposure questionnaire, in which participants were asked to recall exposures in the previous week. A multi-day averaging time was also appropriate for this study since the exact date of exacerbation was defined by the day that patients contacted the nurses to say their symptoms had worsened. Patients may have been sick a few days before they called; the nurses took patients’ calls during normal working hours (7 am-5 pm, Monday through Friday).

Confounding was evaluated by adding variables into the model and observing changes in effect size. Potential confounders were identified a priori in published literature and include meteorological variables (such as temperature and humidity), self-reported information from the exposures questionnaire (such as hours spent outdoors, exercise, respiratory and non-respiratory infections) and season. All confounders were evaluated using the same seven day averaging time as the air pollutants, where applicable.

Two chronic COPD factors were investigated as possible effect modifiers of the association between outdoor air pollution and risk of exacerbation: COPD severity and the presence/absence of asthma as a co-condition. The Global Initiative for Chronic Obstructive Lung Disease (GOLD) defines a widely-used COPD severity classification scheme based on the forced expiratory volume in one second (FEV_1_) [1]. GOLD classifies severity of airflow limitation among COPD patients as “mild” to “very severe” as follows; mild = FEV_1_ ≥ 80 % predicted, moderate = 50 % ≤ FEV_1_ < 80 % predicted, severe = 30 % ≤ FEV_1_ < 50 % predicted, and very severe = FEV_1_ < 30 % predicted [1]. All participants were dichotomously classified with either mild-moderate or severe-very severe COPD using this GOLD classification scheme. A co-condition of asthma was defined using self-reported history of ever receiving a doctor diagnosis of the disease.

Potential non-linear relationships between air pollutant concentrations and COPD exacerbation were evaluated by plotting univariate restricted cubic splines (RCS). RCS provide a flexible nonparametric approach to study the shape of the association, while replacing the linear function of the exposure with a smooth non-linear function [20, 21]. We used the SAS macro “lgtphcurv9”, which implements natural cubic spline methodology to fit potential non-linear response curves in logistic regression models for case-control studies [22]. A number of different splies with varying knots were investigated, with ORs plotted on the y axis and pollutant concentrations on the x axis. All splines were completed while maintaining the match between study subjects and with exposure reference levels set to the first quartile concentration of each pollutant. RCS were also developed for temperature and humidity, with reference values represented by assumed “comfortable” or low risk conditions (such as a temperature of 70 °F). Likelihood ratio tests were performed to test for linear relations and model fit was compared between the linear and spline models.

## Results

A total of 246 COPD patients were enrolled in this study; 75 were subsequently excluded since they did not have at least one exacerbation over the course of the study period and three were excluded because they were hospitalized or in a long-term care facility within the seven days prior to completing one of their exposure questionnaires. The final sample population included 168 COPD patients, contributing information to 231 exacerbation and 389 control periods. In general, these participants were predominantly white, over the age of 65, retired and with severe to very severe COPD (Table 1). A majority of participants were ex-smokers while 20 % still smoked at the start of the study. Nearly half had received a doctor diagnosis of asthma. 65 % of participants experienced one exacerbation over the study period, while 30 % experienced two exacerbations and 5 % experienced three exacerbations.

**Table 1:** Demographics of sample population (*n* = 168 participants)

Age (mean years, SD)	70.1 (9.78)
Female, % (*n*)	60.1 (101)
Race, % (*n*)	
White	97 % (163)
Black	1.2 % (2)
Other	1.8 % (3)
Disease Severity, % (*n*)^a^	
Mild	1.8 % (3)
Moderate	23 % (38)
Severe	50 % (82)
Very Servere	18 % (30)
Normal FEV1/FVC with COPD symptoms	7.1 % (12)
Inadequate spirometry (missing)	1.8 % (3)
Doctor diagnosed asthma, % (*n*)	43 % (73)
Smoking Status, % (*n*)	
Current smoker	19 % (30)
Ex smoker	81 % (131)
Never smoked	4.2 % (7)
Pack Years (mean, SD)	52 (30.89)

^a^Disease severity based on GOLD classification

Average daily NO_2_ concentrations measured in Worcester, Ware and Springfield ranged from 2.79 ppb to 40 ppb, average SO_2_ concentrations ranged from 0.28 ppb to 7.51 ppb and average PM_2.5_ concentrations ranged from 0.45 ug/m^3^ to 37 ug/m^3^ (Table 2). These concentrations are far below current National Ambient Air Quality Standards (NAAQS), set at an annual concentration of 53 ppb for NO_2_, an annual concentration of 12 ug/m^3^ for PM_2.5_, and a one hour concentration of 75 ppb for SO_2_ [7–10]. The mean concentration of PM_2.5_ was 71 % of current NAAQS while the mean concentration of NO_2_ was 21 % of current NAAQS (Table 2). When SO_2_ is compared to the previous 1971 NAAQS, which was based on an annual mean concentration, the yearly mean in our study location was 7 % of the standard. We did not compare to current SO_2_ NAAQS, as this value is now based on a 1 h maximum.

**Table 2:** Summary statistics for air pollutant and meteorological variables

Pollutant or meteorological variable (units)^a^	Minimum	Maximum	Mean ± St. dev	Mean as % of NAAQS^b^
PM_2.5_ (ug/m^3^)	0.45	37.0	8.56 ± 5.4	71 %
NO_2_ (ppb)	2.79	40.0	11.0 ± 5.0	21 %
SO_2_ (ppb)	0.28	7.51	1.99 ± 0.91	7 %
Relative Humidity (%)	22.4	93.9	68.7 ± 13.9	--
Temperature (°F)	0.96	83.8	49.8 ± 17.1	--
Temperature (°C)	−17.2	28.8	9.89 ± −8.3	

^a^Air pollutants: based on daily averages measured in Worcester, Ware and Springfield

Temperature and humidity: based on daily averages from Worcester Regional Airport

^b^Relevant USEPA National Ambient Air Quality Standards (NAAQS)

PM_2.5_ = 12 ug/m^3^ annual mean, averaged over 3 years

NO_2_ = 53 ppb annual mean

SO_2_ = 30 ppb annual mean (based on USEPA 1971 value, which was revoked in 2010 and replaced with a 1-h maximum)

The concentrations of all three pollutants showed typical patterns of higher and more variable concentrations in the winter months (data not shown). Pollutant concentrations were highly correlated with one another in the winter (Table 3), while in spring and summer they were weakly correlated. Pollutant concentrations tended to be poorly correlated with temperature and humidity, with the exception of PM_2.5_ and temperature in the spring (*r* = 0.52) and summer (*r* = 0.62).

**Table 3:** Correlation between air pollutant and meteorological variables

	SO_2_^a^	NO_2_^a^	PM_2.5_^a^	Temperature^b^
NO_2_^a^	Spring: 0.14	--	--	--
	Summer: −0.03			
	Fall: 0.52**			
	Winter: 0.80**			
PM_2.5_^a^	Spring: 0.30**	Spring: 0.41**	--	--
	Summer: 0.20*	Summer: 0.45**		
	Fall: 0.39**	Fall: 0.73**		
	Winter: 0.79**	Winter: 0.83**		
Temperature^b^	Spring: 0.34**	Spring: 0.02	Spring: 0.52**	--
	Summer: 0.24**	Summer: 0.15*	Summer: 0.62**	
	Fall: 0.02	Fall: −0.04	Fall: −0.15*	
	Winter: −0.13	Winter: 0.09	Winter: 0.11	
Relative Humidity^b^	Spring: 0.09	Spring: 0.07	Spring: −0.01	Spring: −0.25**
	Summer: −0.13	Summer: 0.02	Summer: −0.07	Summer: 0.22**
	Fall: −0.14*	Fall: −0.24**	Fall: −0.28**	Fall: 0.38**
	Winter: −0.02	Winter: 0.03	Winter: 0.08	Winter: 0.22**

Values shown are Pearson Correlation Coefficients; * indicates *p* < 0.05 and ** indicates *p* <0.0001

^a^24-h average concentrations; Worcester, Ware and Springfield monitors from 2011 to 2012

^b^24-h average measurements; Worcester Regional Airport meteorological site from 2011–2012

**Fig. 1:**
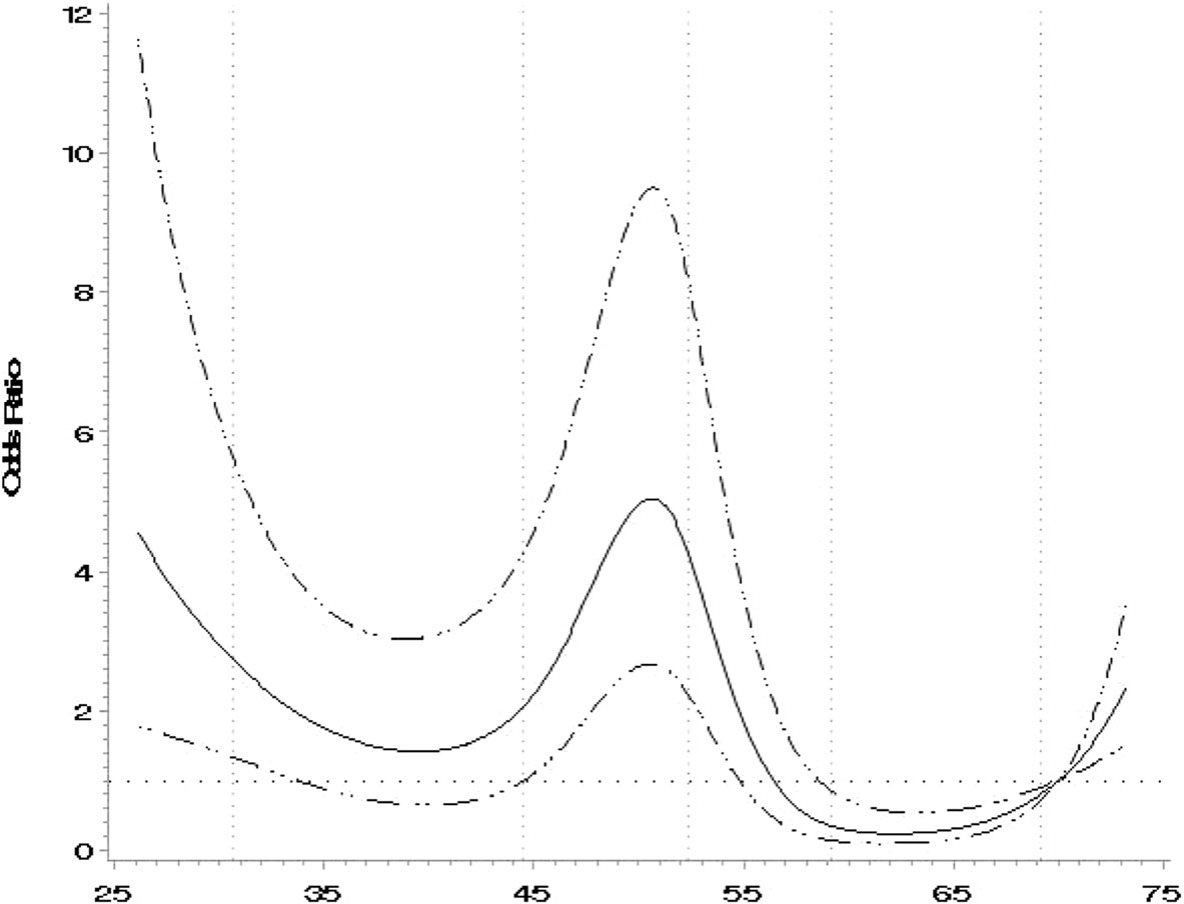
Restricted Cubic Spline for Temperature. Notes: Reference exposure value set at 70 °F, including 5 knots. This figure displays a restricted cubic spline from conditional logistic regression with temperature as the predictor and COPD exacerbation as the outcome

Self-reported exposure to cold temperatures in the previous week modestly increased the risk of exacerbation (OR = 1.16, 95 % CI: 0.78–1.70), while self-reported exposure to hot temperatures decreased the risk of exacerbation (OR = 0.50, 95 % CI: 0.34–0.75). When temperature was evaluated as a continuous linear term, using the 1–7 day average of measurements recorded at Worcester Regional Airport, a weak negative association was observed (OR = 0.96, 95 % CI: 0.95–0.98), further suggesting a decreased risk of exacerbation with increasing temperatures. Similar effects were observed when using the 1–2 day and 1–3 day averages of temperature as linear terms. The RCS, however, identified a markedly non-linear relationship between temperature and exacerbation, and showed considerable improvement in model fit over the linear model (−2LL = 384 and 443 for RCS and linear model, respectively) (Fig. 1). The spline was therefore used to create a trichotomous variable with cut points at the approximate inflection points of 40 °F and 50 °F (4.4 °C and 10 °C). When this categorical representation of temperature was included in the conditional logistic regression model with cold-cool temperatures set as the reference group, a strong positive association was observed for moderate temperatures and exacerbation (OR = 2.50, 95 % CI: 1.51–4.12), while a strong negative association was found for the warm-hot temperatures and exacerbation (OR = 0.55, 95 % CI: 0.36–0.81).

Humidity was only modestly correlated with temperature (*r* = 0.27), but exhibited a similar non-linear relationship with risk of exacerbation. Analogous to the approach just described for temperature, a dichotomous relative humidity variable was created from the spline (not shown), which indicated a logical cut point at 65 % relative humidity. The linear and dichotomous relative humidity variables did not fit the COPD exacerbation risk as well as temperature, and so humidity was not further evaluated. Strong effects were also observed for season, with the strongest risk of COPD exacerbation in the fall (OR = 6.64, 95 % CI: 3.83–11.5), followed by spring (OR = 2.59, 95 % CI: 1.55–4.35) and winter (OR = 2.38, 95 % CI: 1.37–4.11). Since temperature was highly correlated, season was not further evaluated.

Several different variables were investigated to study effects of respiratory infections on COPD exacerbation including: self-reported respiratory infection, non-respiratory infection, and sinus infection, as well as regional influenza trends (Table 4). The strongest association, by far, was found for self-reported respiratory infections (OR = 8.14, 95 % CI: 4.67–14.2). This effect was twice as strong as that estimated with CDC’s indicator of flu (OR = 3.78, 95 % CI: 1.54–9.28), suggesting stronger effects when using individual level rather than population level data.

**Table 4:** Non-chemical triggers and COPD exacerbation (Weather, flu, and physical activity)

Variable type	Variable description	Odds ratio	95 % confidence interval		−2loglikelihood
Weather	Spent time in cold air/cold weather in past week^a^	1.16	0.78	1.70	468
	Spent time in hot air/hot weather in past week^a^	0.50	0.34	0.75	457
	Local Temperature (linear 7 day average)^b^	0.96	0.95	0.98	445
	Local Temperature (categorical)^c^				
	Cool/Cold	1.0	1.0	1.0	422
	Moderate	2.50	1.51	4.12	
	Warm/Hot	0.55	0.36	0.84	
	Local Relative Humidity (linear 7 day average)^b^	0.98	0.96	1.01	471
	Local Relative Humidity (categorical)^c^				
	High	1.0	1.0	1.0	471
	Low	1.21	0.84	1.73	
Season	Season, *ref = summer*				
	Summer	1.0	1.0	1.0	417
	Spring	2.59	1.55	4.35	
	Winter	2.38	1.37	4.11	
	Fall	6.64	3.83	11.5	
Acute Illness	Respiratory infection/cold in past week^a^	8.14	4.67	14.2	396
	Non-respiratory Illness in past week^a^	1.65	0.67	4.05	422
	Sinus infection in past week^a^	2.55	1.20	5.44	461
	Regional Flu Trends; New England^d^	3.78	1.54	9.28	463
Physical Activity	Total hours spent outside of the home^a, e^	0.99	0.99	1.00	469
	Exercised in past week^a^	0.60	0.39	0.93	462

^a^Based on self-reports from exposure questionnaires

^b^Based on 7-day averages using data from NOAA collected at Worcester Regional Airport

^c^Categorical representation of variable based on cut-offs identified in restricted cubic splines

^d^Regional proxy of flu from CDC data; represented by percent of weekly outpatient visits for influenza-like-illness in New England

^e^Estimated as sum of “Hours Reported Outdoors”, “Hours Reported in a Vehicle”, and “Hours Reported Indoors but not at home”

Self-reported exercise and hours spent outdoors were negatively associated with COPD exacerbation (Table 4). This may be because those who exercised and spent time outdoors were healthy enough to do so and therefore less likely to exacerbate. It may also be that those soon to exacerbate were aware of the impending exacerbation and adjusted their behavior to intentionally avoid more strenuous activities.

In single-pollutant models, SO_2_ and NO_2_ were found to be positively associated with COPD exacerbation, OR = 2.78 (95 % CI: 2.08–3.70) and OR = 1.17 (95 % CI: 1.09–1.25), respectively. Effect estimates were slightly weakened for SO_2_ (OR = 2.28 (95 % CI: 1.64–3.19) and NO_2_ (OR = 1.06 (95 % CI: 0.98–1.16) when temperature and self-reported respiratory infection were included in the model, suggesting some confounding by these latter variables. Unexpectedly, a small negative effect for PM_2.5_ and COPD exacerbation was identified (OR = 0.93 (95 % CI: .088–0.99), which was unaffected by temperature and self-reported respiratory infection (Table 5). Self-reported hours outdoors and exercise did not confound these associations or improve model fit.

**Table 5:** Single and multi-air pollutant models

Model	Without adjustment					With adjustment^b^				
	OR (95 % CI)^a^				-2 log likelihood	OR (95 % CI)^a^				-2 log likelihood
Single Pollutant Models										
1. NO_2_	1.17	1.09	-	1.25	451	1.06	0.98	-	1.16	360
2. SO_2_	2.78	2.08	-	3.70	413	2.28	1.64	-	3.19	336
3. PM_2.5_	0.93	0.88	-	0.99	467	0.94	0.88	-	1.01	359
Multi-Pollutant Models										
4. NO_2_	1.04	0.96	-	1.12	412	0.96	0.88	-	1.06	335
SO_2_	2.58	1.87	-	3.55		2.43	1.69	-	3.50	
5. NO_2_	1.31	1.21	-	1.43	423	1.17	1.05	-	1.30	351
PM_2.5_	0.81	0.74	-	0.88		0.87	0.79	-	0.96	
6. SO_2_	2.99	2.22	-	4.02	403	2.45	1.75	-	3.45	329
PM_2.5_	0.90	0.84	-	0.96		0.91	0.85	-	0.98	
7. NO_2_	1.16	1.05	-	1.27	393	1.04	0.93	-	1.17	329
SO_2_	2.37	1.71	-	3.28		2.33	1.62	-	3.37	
PM_2.5_	0.84	0.77	-	0.91		0.89	0.81	-	0.98	

^a^Odds Ratios (OR) represent a one unit increase in pollutant concentration (ppb or ug/m^3^)

^b^Adjusted for temperature (categorical representation based on cut-offs from RCS) and self-reported respiratory infection

A positive linear association for both SO_2_ and NO_2_ and risk of COPD exacerbation was seen in univariate restricted cubic splines (tests for linearity yielded *p* values < 0.0001) while a negative linear association was observed for PM_2.5_ and COPD exacerbation (*p* = <0.0001). For all three pollutants, linear and RCS models had comparable fits (−2LL improvement of less than 1 %), suggesting that the use of a flexible smoothing splines did not improve upon the more parsimonious linear models.

In order to gain insight into which individual pollutants might influence COPD exacerbation independently of the effects of other pollutants, multi-pollutant models were explored (Table 5). In multi-pollutant conditional logistic regression models, effect estimates were stronger and model fit improved for SO_2_ and NO_2_ after adjustment for PM_2.5_, and vice versa. When both SO_2_ and NO_2_ were included in models, the positive effect observed for NO_2_ was removed entirely. When all three pollutants were modeled together, stronger positive effects were observed for SO_2_ and stronger negative effects were observed for PM_2.5_; there was no impact on NO_2_.

Based on these findings, a final predictive model was developed, which included SO_2_, PM_2.5_, and the two identified confounders (temperature and self-reported respiratory infection). NO_2_ was not included since inclusion of this pollutant did not improve model fit or have any impact on the pollutants, when including SO_2_ and PM_2.5_. In this model, SO_2_ showed a strong positive effect (OR = 2.45, 95 % CI: 1.75–3.45) and PM_2.5_ showed a weak negative effect (OR = 0.91, 95 % CI: 0.85–0.98). Both self-reported respiratory infection (OR = 6.70, 95 % CI: 3.64–12.3) and temperature (moderate temperature OR = 2.45, 95 % CI: 1.37–4.38 and warm-hot temperature OR = 0.95, 95 % CI: 0.57–1.57) also showed strong effects.

As noted in the methods section, we also investigated the risk of COPD exacerbation associated with self-reports of common exposures experienced in everyday life (i.e., air fresheners, barbequing, raking leaves, etc.). We found that car and truck exhaust and scented laundry products were associated with increased risk of exacerbation (Table 6 in Appendix). These associations, which are explored further in a separate publication currently in preparation, did not confound any of the associations between air pollutants and COPD exacerbation.

Nearly half (43 %) of the participants reported that they had physician-diagnosed asthma as well as COPD. A similar extent of co-occurrence (this has been termed Asthma and COPD Overlap Syndrome (ACOS)) appears to be common; a recent meta-analysis found the prevalence of ACOS ranging from 13 to 56 % among 9 published population based COPD studies [23]. Our study was not designed to investigate differences in air pollution effects in the presence or absence of an asthma diagnosis, but with the available data we attempted to evaluate the hypothesis that the presence of asthma as a co-morbid condition might make COPD patients more susceptible to risk of exacerbation from air pollutants. In models stratified on asthmatic status, the only notable difference observed was that NO_2_ appeared to have a somewhat stronger effect among non-asthmatics (OR = 1.14, 95 % CI: 0.97–1.34) compared to asthmatics (OR = 0.94 (0.78–1.13)), although the smaller sample size in these subgroups resulted in wide confidence intervals. In an analogous way, we evaluated the hypothesis that disease severity might modify response to air pollution. Comparing mild and moderate to severe and very severe COPD in the GOLD classification, there did not appear to be important differences in the strength of the associations between any of the air pollutants studied and COPD exacerbation. We repeated all analyses after omitting the 7 % of subjects who had normal FEV1/FVC with COPD symptoms (Table 1); there were no important differences in the results.

## Discussion

This study provides evidence for effects of short term exposures to relatively low levels of outdoor air pollution on exacerbation of existing COPD among patients residing in central Massachusetts. The study found strong positive associations between short-term exposures to outdoor SO_2_ and NO_2_ and COPD exacerbation. The estimate of the effect of NO_2_ was reduced, however, in the presence of SO_2_ suggesting confounding or that the two gaseous pollutants may compete with one another in regression models. Risk estimates for both pollutants were considerably larger and found at lower concentrations than reported in other studies of COPD-related ED visits, HA, and mortality [4]. There are several possible explanations for these findings. First, our study population of COPD patients enrolled in a DMG and the case-crossover design may have enabled us to detect earlier and milder cases of exacerbation that would not have resulted in ED visits or HA. Indeed, the goal of the DMG is precisely to intervene early in the development of exacerbations to prevent these costly outcomes. This is demonstrated by the fact that only seven of the 231 exacerbations captured in this study resulted in a hospitalization. Also, the close monitoring of patients by study nurses made it possible to economically and fairly non-intrusively gather data from patients at random times when they were not experiencing exacerbations (control periods), which provided essential comparison data with which to compare the exposures immediately prior to an exacerbation.

It is also possible that these findings were due to chance or an un-identified bias. For example, the measured pollutants may actually represent surrogates for another source of air pollution in this region, and the findings may be an artifact of measurement error at low level concentrations. Measurement error is especially problematic for SO_2_, since concentrations in this region are often near USEPA’s level of detection (LOD), concentrations for which there is well documented measurement uncertainty [9]. On the other hand, these results were robust upon adjustment by many different potential confounders, including other criteria pollutants and meteorological variables. It is possible that stronger effects were observed here due to the more sensitive method of identifying exacerbations or since this study was able to control for important confounders on the individual level, by design. This is one important advantage of the case-crossover study; covariates which are either fixed or change only very slowly with time cannot confound the observed associations. Among these are age, gender, smoking, and occupational exposure history.

An unexpected negative association was found for short-term exposures to outdoor PM_2.5_ and COPD exacerbation. While this is inconsistent with a majority of the published literature concerning air pollution and COPD-related ED-HA, there are a number of possible explanations. The chemical composition and therefore toxicity of PM is highly variable across geographic areas and through time, making it difficult to compare results from studies conducted in different regions and in different seasons/years. For example, PM constituents in the eastern US are known to have higher sulfate concentrations than much of the rest of the country [8]. Furthermore, in the eastern US, the sulfate component of PM is higher in the summer, when oxidation of SO_2_ occurs at a faster rate than in the winter [8]. PM components are also known to vary by temperature, relative humidity, and wind speed [24]. A review of PM speciation data for USEPA fixed site monitors located in Chicopee, MA (adjacent to Springfield) and Ware, MA demonstrated the complex temporal and spatial nature of PM chemistry. In brief, the ratios of sulfates and sulfur to total PM_2.5_ mass concentration were relatively stable across the four seasons but consistently higher in the urban area of Chicopee than in rural area of Ware. The ratios of nitrates to total PM, on the other hand, were similar at both locations but nearly two-folder higher in winter and fall than in the spring and summer.

It is also possible our understanding of the effects of PM2.5 may need to be adjusted to reflect long term changes in air pollution levels. USEPA estimates that PM_2.5_ concentrations dropped 10 % between 1999 and 2007 [8], and as pollutant concentrations continue to decline and source contributions change, results estimated from studies published across decades may not be comparable in the same way that results across geographic areas and seasons may not be comparable.

The unexpected results for PM_2.5_ may also be due to the selected exposure window. A majority of published literature has evaluated shorter time periods of exposure (such as one or two days) even though EPA recommends including longer time spans when evaluating associations between PM and respiratory outcomes, in their ISA documents [8]. Longer lag/averaging times are considered biologically plausible due to PM effects on allergic sensitization and lung immune defenses, which have been observed in controlled human exposure and animal toxicological studies [8]. Still, it is not clear whether the seven-day window that we chose to use was appropriate and whether an even longer exposure period may have been more suitable. When exploring temporal variation in our sensitivity analyses, evidence for air pollutant effects on COPD exacerbation was observed as far as thirty days prior to the health event (data not shown).

Finally, these unexpected results raise the interesting question of publication bias, which will occur if the published literature is systematically unrepresentative of the population of studies that were actually completed [25]. If other studies have been completed in areas of similar concentrations but not published due to weak, null, negative or “inconsistent” findings, it could lead to the false conclusion that our results are inconsistent with the general state of the science.

Consistent with existing literature, this study found that temperature and season were associated with COPD exacerbation, independent of air pollution, with higher risks in colder seasons [26, 27]. This study also found that low humidity increased the risk COPD exacerbation while higher humidity appeared protective, although confidence intervals were wide. Similar findings were reported by Qui and colleagues in a recent time series study that evaluated the impact of season and humidity on COPD hospitalizations in Hong Kong [28]. They reported stronger effects on cold and dry days, hypothesizing that humid conditions protect mucous membranes against toxic insults [28].

Since temperature and humidity are independent risk factors for COPD exacerbation but also major drivers of day-to-day fluctuations of air pollution concentrations, teasing apart the “independent” effects of air pollution and climate can be difficult. Correlations among pollutants and meteorological variables varied considerably across seasons and therefore models fit to data for an entire year may retain important residual confounding or result in over-fitting for some seasons [29].

This study quantified air pollution exposures using secondary data collected at USEPA fixed central monitors, covering the catchment area of the study population. This introduced the potential for exposure misclassification, which may have biased results. This includes exposure misclassification due to; 1) analytical measurement error (which likely varies across pollutants and by season); 2) day-to-day variability at the monitor versus day-to-day variability in the larger geographic region; and 3) discrepancies between personal exposures and outdoor air pollution concentrations. We expect that the misclassification from these sources was likely nondifferential with respect to COPD exacerbations and thus on average would be expected to result in an underestimation of the true association.

Although the case crossover study design has many advantages when studying acute outcomes, the matched nature of this design could have introduced some bias. The case crossover design is limited in its ability to explore effect modification by variables that change through time (such as season, temperature, and humidity). It is possible that we might have found different results, particularly for PM_2.5_, if we were able to look at risk separately within the different seasons. This is especially true since the air pollution effect estimates were small relative to the estimated effects of season. It is also possible that we might have observed different effects if we had bidirectional control sampling, where control periods were assigned shortly before and after exacerbation within the same season [30, 31]. However, this design is difficult in practice for a debilitating outcome like COPD exacerbation, which can last up to a month.

## Conclusions

This study found consistent positive effects of SO_2_ and modest negative effects of PM_2.5_ on COPD exacerbation, at concentrations below current USEPA NAAQS. The magnitude of risk of COPD exacerbation from exposure to outdoor SO_2_ was generally higher than reported in other studies. This study also found a positive effect of NO_2_; however, this effect was only significant when included in models with PM_2.5_.

If supported by additional studies with better exposure assessments, these findings may suggest risk of COPD exacerbation at lower air pollution concentrations than reported for COPD-related ED, HA, and mortality. Characterization of air pollution in the context of COPD exacerbation studied here (experienced in the persons’ home and therefore not necessarily requiring a visit to the emergency room or hospital) should be conducted in other locations with different sources of pollutants and climate. This would improve our understanding of the complex relationships between air pollutant and meteorological variables and their influence on risk of COPD exacerbation.

## Appendix

**Table 6:** Final model including car/truck exhaust and scented laundry

Variable	Odds ratio	95 % confidence interval	
SO_2_^a^	2.61	1.84	3.74
PM_2.5_^a^	0.92	0.85	0.99
Temperature^b^			
Cool/Cold	1.0	1.0	1.0
Moderate	2.31	1.26	4.25
Warm/Hot	0.78	0.46	1.31
Respiratory Infection/Cold^c^	7.53	3.95	14.4
Car/Truck Exhaust^c^	2.78	1.31	5.93
Scented Laundry^c^	4.88	1.84	12.9

^a^Based on 7-day averages using data from USEPA collected in Springfield, Ware and Worcester

^b^Based on 7-day averages using data from NOAA collected at Worcester Regional Airport

^c^Based on self-reports of exposures in previous week; collected from questionnaires

## Abbreviations

AQS: Air quality system
CDC: Center for Disease Control
COPD: Chronic obstructive pulmonary disease
DMG: Disease management group
ED: Emergency department visit
FEV_1_: Forced expiratory volume in one second
GOLD: Global Initiative for Chronic Obstructive Lung Disease
HA: Hospitalization
IliNet: Influenza like illness surveillance network
LOD: Level of detection
NAAQS: National Ambient Air Quality Standards
NCDC: National Climatic Data Center
NOAA: National Oceanic and Atmospheric Administration
NO2: Nitrogen dioxide
OR: Odds ratio
O: Ozone
PM_10_: Particulate matter
PM_2.5_: Particulate matter
ppb: Parts per billion
SO2: Sulfur dioxide
RCS: Restricted cubic splines
ug/m^3^: Microgram per cubic meter
2LL: −2 log likelihood

## References

[1] Global Initiative for Chronic Lung Disease (GOLD). Global Strategy for Diagnosis, Management and Prevention of Chronic Obstructive Pulmonary Disease. 2014.

[2] White A, Gompertz S, Stockley R (2003). Chronic obstructive pulmonary disease: the etiology of exacerbations of chronic obstructive pulmonary disease. Thorax Review Series.

[3] Institute for Quality and Efficiency in Health Care (IQEHC), Chronic coughing and breathing difficulties: Chronic obstructive pulmonary disease (COPD) Available: http://www.ncbi.nlm.nih.gov/pubmedhealth/PMH0005192/. 2011. Accessed May 2013.

[4] DeVries R. Short term effects of outdoor air pollution on respiratory health in susceptible populations (Doctoral dissertation). University of Massachusetts Lowell. Retrieved from ProQuest Dissertations and Theses. (Order No. 2662940), 2015.

[5] Ko F, Hui D (2012). Air pollution and chronic obstructive pulmonary disease. Respirology.

[6] Ferrari U, Exner T, Wanka E (2012). Influence of air pressure, humidity, solar radiation, temperature and wind speed on ambulatory visits due to chronic obstructive pulmonary disease in Bavaria, Germany. Int J Biometerol.

[7] United States Environmental Protection Agency (USEPA). Criteria Air Pollutants. 2016. https://www.epa.gov/criteria-air-pollutants. 2016. [Accessed 30 Aug 2016].

[8] USEPA, Integrated Science Assessment for Particulate Matter*.* EPA/600/R-08/139 F. National Center for Environmental Assessment. RTP Division, Research Triangle Park, NC. December 2009.

[9] USEPA, Integrated Science Assessment for Sulfur Oxides - Health Criteria. EPA/600/R-08/047 F, National Center for Environmental Assessment. RTP Division, Research Triangle Park, NC. September 2008.

[10] USEPA, Integrated Science Assessment for Oxides of Nitrogen - Health Criteria*.* EPA/600/R-08/071, National Center for Environmental Assessment. RTP Division. Research Triangle Park, NC. July 2008.

[11] Desqueyroux H, Pujet J, Prosper M, Moullec YL, Momas I (2002). Effects of air pollution on adults with chronic obstructive pulmonary disease. Arch Environ Health.

[12] Peacock JL, Ross Anderson H, Strachan DP, Bremner SA, Marston L, Seemungal TA, Wedzicha JA (2011). Outdoor air pollution and respiratory health in patients with COPD. Thorax.

[13] Maclure M (1991). The case-crossover design: A method for studying transient effects on the risk of acute events. Am J Epidemiol.

[14] Maclure M, Mittleman MA (2000). Should we use a case-crossover design?. Annu Rev Public Health.

[15] City of Worcester: Demographics and Census Information. Available: http://www.worcestermass.org/home/about-worcester/special-events/demographics-censusinformation. 2014. Accessed Mar 2014.

[16] USEPA: Technology Transfer Network (TTN): Air Quality System (AQS). Available: https://www.epa.gov/aqs. 2014. Accessed Jan 2014.

[17] USEPA 2014, PM_2.5_ Continuous Monitor Comparability Assessment. Available: http://www.epa.gov/airquality/airdata/ad_rep_frmvfem.html. 2014. Accessed Jan 2014.

[18] National Oceanic and Atmospheric Administration (NOAA), 2013. National Climatic Data Center. Available: http://www.ncdc.noaa.gov/. 2013. Accessed Jan 2014.

[19] Center for Disease Control and Prevention (CDC). 2014 U.S. Influenza Surveillance Report. http://www.cdc.gov/flu/weekly/. (2014). Accessed Apr 2014.

[20] Govindarajulu US, Malloy EJ, Ganguli B, Spiegelman D, Eisen EA (2009). The comparison of alternative smoothing methods for fitting non-linear exposure-response relationships with Cox models in a simulation study. Int J Biostat.

[21] Govindarajulu US, Spiegelman D, Thurston SW, Ganguli B, Eisen EA (2007). Comparing smoothing techniques in Cox models for exposure-response relationships. Stat Med.

[22] Li R, Hertzmark E, Louie M, Chen L, Spiegelman D. The SAS LGTPHCURV9 Macro 2010.

[23] Alshabanat A, Zafari Z, Albanyan O, Dairi M, FitzGerald JM (2015). Asthma and COPD Overlap Syndrome (ACOS): A Systematic Review and Meta-Analysis. PLoS One.

[24] Tai APK, Mickley LJ, Jacob DJ (2010). Correlations between fine particulate matter (PM2.5) and meteorological variables in the United States: implications for the sensitivity of PM2.5 to climate change. Atmos Environ.

[25] Rothstein H, Sutton A, Borenstein M (2005). Publication Bias in Meta-Analysis, Prevention, Assessment and Adjustments.

[26] Jenkins CR, Celli B, Ferguson GT, Yates JC, Anderson JA, Jones PW, Vestbo J, Calverley PMA (2012). Seasonality and determinants of moderate and severe COPD exacerbations in the TORCH study. Eur Respir J.

[27] Donaldson GC, Goldring JJ, Wedzicha JA (2012). Influence of season on exacerbation characteristics in patients with COPD. Chest.

[28] Qiu H, Yu ITS, Wang X, Tian L, Tse LA, Wong TW (2013). Season and humidity dependence of the effects of air pollution on COPD hospitalizations in Hong Kong. Atmos Environ.

[29] Ito K, Thurston GD, Silverman RA (2007). Characterization of PM2.5, gaseous pollutants, and meteorological interactions in the context of time-series health effects models. J. Expo. Sci. Environ. Epidemiol..

[30] Janes H, Sheppard L, Lumley T (2005). Case-crossover analyses of air pollution exposure data: referent selection strategies and their implications for bias. Epidemiology.

[31] Bateson TF, Schwartz J (2001). Selection bias and confounding in case crossover analyses of environmental time-series data. Epidemiology.

